# Lipoprotein lipase expression, serum lipid and tissue lipid deposition in orally-administered glycyrrhizic acid-treated rats

**DOI:** 10.1186/1476-511X-8-31

**Published:** 2009-07-29

**Authors:** Wai Yen Alfred Lim, Yoke Yin Chia, Shih Yeen Liong, So Ha Ton, Khalid Abdul Kadir, Sharifah Noor Akmal Syed Husain

**Affiliations:** 1 School of Science, Monash University Sunway Campus, Jalan Lagoon Selatan, Bandar Sunway 46150, Selangor Darul Ehsan, Malaysia; 2 School of Medicine and Health Sciences, Monash University Sunway Campus, Jalan Lagoon Selatan, Bandar Sunway 46150, Selangor Darul Ehsan, Malaysia; 3 Cytopathology and Cytogenetics Unit, Department of Pathology, Universiti Kebangsaan Malaysia Medical Centre, Jalan Yaacob Latif, Bandar Tun Razak, Cheras 56000, Kuala Lumpur, Malaysia

## Abstract

**Background:**

The metabolic syndrome (MetS) is a cluster of metabolic abnormalities comprising visceral obesity, dyslipidaemia and insulin resistance (IR). With the onset of IR, the expression of lipoprotein lipase (LPL), a key regulator of lipoprotein metabolism, is reduced. Increased activation of glucocorticoid receptors results in MetS symptoms and is thus speculated to have a role in the pathophysiology of the MetS. Glycyrrhizic acid (GA), the bioactive constituent of licorice roots (*Glycyrrhiza glabra*) inhibits 11β-hydroxysteroid dehydrogenase type 1 that catalyzes the activation of glucocorticoids. Thus, oral administration of GA is postulated to ameliorate the MetS.

**Results:**

In this study, daily oral administration of 50 mg/kg of GA for one week led to significant increase in LPL expression in the quadriceps femoris (*p *< 0.05) but non-significant increase in the abdominal muscle, kidney, liver, heart and the subcutaneous and visceral adipose tissues (*p *> 0.05) of the GA-treated rats compared to the control. Decrease in adipocyte size (*p *> 0.05) in both the visceral and subcutaneous adipose tissue depots accompanies such selective induction of LPL expression. Consistent improvement in serum lipid parameters was also observed, with decrease in serum free fatty acid, triacylglycerol, total cholesterol and LDL-cholesterol but elevated HDL-cholesterol (*p *> 0.05). Histological analysis using tissue lipid staining with Oil Red O showed significant decrease in lipid deposition in the abdominal muscle and quadriceps femoris (*p *< 0.05) but non-significant decrease in the heart, kidney and liver (*p *> 0.05).

**Conclusion:**

Results from this study may imply that GA could counteract the development of visceral obesity and improve dyslipidaemia via selective induction of tissue LPL expression and a positive shift in serum lipid parameters respectively, and retard the development of IR associated with tissue steatosis.

## Background

Lipoprotein lipase (LPL) is the major enzyme responsible for the hydrolysis of circulating triacylglycerol (TAG) moiety of both classes of TAG-rich lipoproteins; the chylomicrons and very-low-density lipoprotein (VLDL), generating free fatty acids (FFA) that are either oxidized in the muscles or re-esterified in the adipose tissues, and glycerol that is returned to the liver. LPL plays a central role in overall lipoprotein metabolism, where (i) the successive interaction of VLDL with LPL generates the low-density lipoproteins (LDL) that are involved in forward cholesterol transport and (ii) the remnant lipoprotein particles so formed from LPL catalysis contributes to the maturation of high-density lipoprotein (HDL) precursors, the latter of which is then involved in reverse cholesterol transport [1,2]. Perturbation in LPL activity could therefore lead to significant metabolic consequences and LPL has been implicated in pathophysiological conditions characterized by marked hypertriglyceridaemia, such as that observed in the metabolic syndrome (MetS).

The MetS refers to a constellation of metabolic abnormalities characterized by the co-existence of insulin resistance (IR), visceral obesity, hyperglycaemia, hypertension and dyslipidaemia. The syndrome has become a recognizable clinical cluster of risk factors that are predictive of the progression to cardiovascular disease and type 2 diabetes mellitus (T2DM) [3]. Both visceral obesity and IR are recognized as the major determinants in the development of the MetS [4] and in fact, over 80% of individuals with T2DM are obese and virtually all are insulin resistant [5]. Differing definitions of the syndrome have been put forward by various global health agencies such as the World Health Organization (WHO), the National Cholesterol Education Program Adult Treatment Panel III (NCEP ATP III) and the International Diabetes Federation (IDF) but all such definitions point to a common agreement that the syndrome results in increased atherogenesis and death from myocardial infarction [4]. Thus, increased attention has been channeled to the improvement of lipid abnormalities characteristic of the MetS.

Dyslipidaemia, the hallmark of the MetS which is manifested in the more severe form in T2DM, is characterized by (i) increased flux of FFA, (ii) elevated TAG level (hypertriglyceridaemia), (iii) reduced HDL level and (iv) a predominance of small, dense LDL. Elevated plasma FFA is viewed as the primary defect leading to the development of dyslipidaemia [6,7] and IR [8]. With the ensuing IR, LPL expression is reduced and LPL activity becomes diminished [9,10]. This amplifies the extent of the hypertriglyceridaemia by favouring the accumulation of TAG-rich chylomicrons and VLDL in the circulation. The increase in small, dense LDL and low HDL is secondary to this elevated TAG level, where through the action of cholesteryl ester transfer protein (CETP), TAG enrichment of both the HDL and LDL particles occurs. TAG-rich LDL particles are good substrate to be acted upon by hepatic lipase (HL), producing a population of small, dense, lipid-poor LDL. Similarly, HL-mediated hydrolysis of TAG-rich cholesterol-poor HDL leads to an accelerated degradation of apo A-I, the major protein of HDL. This causes the HDL to be rapidly cleared from the plasma [6,7,11]. In addition to such serum lipid perturbations, studies have also indicated that tissue lipid accumulation is associated with obesity-related IR and T2DM where both conditions are associated with increased tissue lipid [12,13].

Increased activation of glucocorticoid receptors has been implicated in the development of MetS symptoms such as visceral obesity and hyperlipidemia. Pharmacological inhibition of the enzyme 11β-hydroxysteroid dehydrogenase type 1 (11β-HSD1) that acts to regenerate active glucocorticoids from inactive 11-keto metabolites has been proposed as a therapeutic target for the treatment of MetS following the association of such inhibition with a cardioprotective lipid profile [14,15]. Glycyrrhizic acid (GA), the primary bioactive constituent of the roots of the shrub *Glycyrrhiza glabra *and its pharmacologically active metabolite glycyrrhetic acid (GE) act as potent, non-selective inhibitors of both isoforms of 11β-HSD [16,17]. To date however, the effects of orally-administered GA on LPL expression and on the modulation of serum lipid and tissue lipid deposition have yet to be conducted. The objectives of this study are therefore to determine and compare each of these parameters between GA-treated and non-treated rats following daily oral administration of GA for one week in the former.

## Results

### GA treatment led to increase in LPL expression of all studied tissues

**Figure 1:**
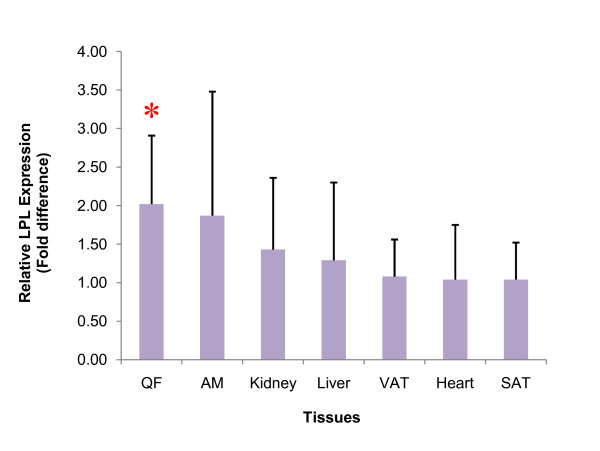
**Fold difference in tissue LPL expression of the GA-treated group**. Relative tissue LPL expression following GA treatment is shown in decreasing order. In this analysis, β-actin (BAC) gene was used as the endogenous reference, GA-treated group as the target and control group as the calibrator. * denotes *p *< 0.05.

LPL expression in the GA-treated rats was increased in all studied tissues (Figure 1), of which included the heart, liver, kidney, quadriceps femoris (QF), abdominal muscle (AM), visceral adipose tissue (VAT) and subcutaneous adipose tissue (SAT). The QF demonstrated the highest increase with a fold difference of 2.02 ± 0.89, representing a significant 102% increase (*p *< 0.05). This was followed by the AM (1.87 ± 1.61 fold; 87% increase), kidney (1.43 ± 0.93 fold; 43% increase), liver (1.29 ± 1.01 fold; 29% increase) and the VAT (1.08 ± 0.48 fold; 8%); all of which exhibited no significance difference between the control and GA-treated group (*p *> 0.05). Increase in LPL expression was similar in the heart and SAT (1.04 ± 0.48; 4% increase) but these were not significant (*p *> 0.05).

### GA treatment reduced the size of adipocytes

**Figure 2:**
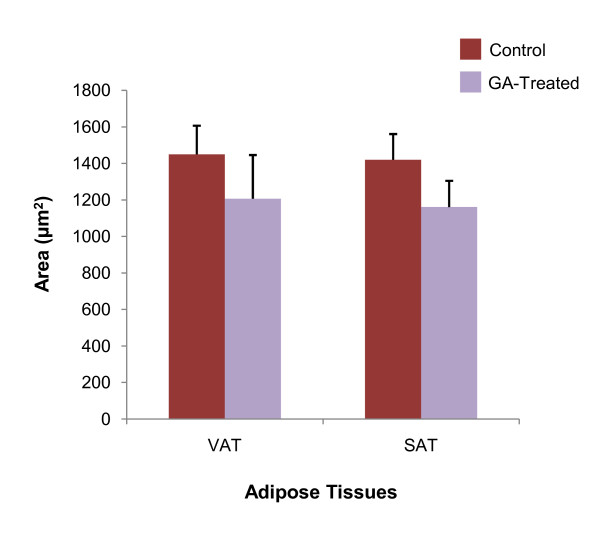
**Mean area of adipocytes (μm^2^) of control and GA-treated rats**. Size of adipocytes demonstrated a decrease in both the VAT and SAT depot after seven days of oral GA administration (*p *> 0.05).

**Figure 3:**
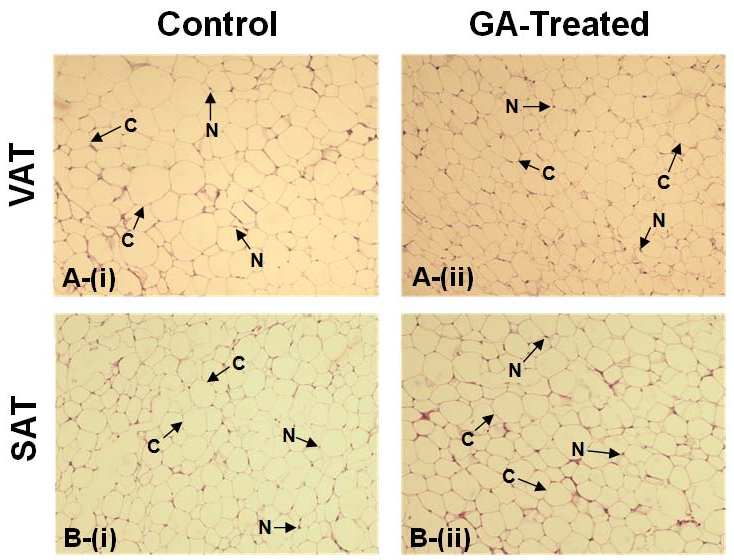
**H&E-stained adipose tissues**. Representative sections of H&E-stained (A) VAT and (B) SAT in (i) control and (ii) GA-treated rats at 100× magnification. The adipocytes appear as empty, unstained vacuoles with the nucleus compressed to one side of the cell while the cytoplasm is reduced to only a small rim at the periphery of the cell. Arrows indicate examples of cytoplasm (C) and nucleus (N).

Mean area of both VAT and SAT adipocytes showed non-significant decrease in the GA-treated group compared to the control (*p *> 0.05) (Figure 2). In the VAT, mean adipocyte area in the control group was 1449.96 ± 156.58 μm^2 ^while that in the treated group was 1206.58 ± 239.48 μm^2^. In the SAT, mean adipocyte area was 1419.91 ± 141.14 μm^2 ^in the control group, compared to a mean of 1161.18 ± 143.26 μm^2 ^in the treated group. These represented a 16.79% and 18.22% reduction in the area of adipocytes in VAT and SAT respectively. Sections of these tissues are shown in Figure 3.

### GA treatment led to improvement in all serum lipid parameters

**Figure 4:**
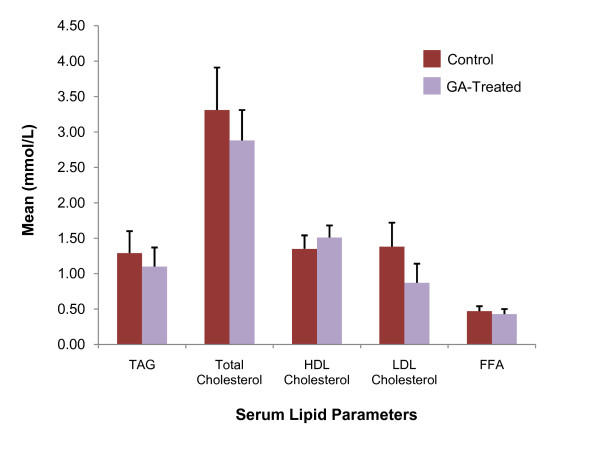
**Serum lipid of control and GA-treated rats**. Mean serum TAG, total cholesterol, LDL-cholesterol and FFA (mmol/L) of GA-treated rats showed reduction after seven days of oral GA administration while that of HDL-cholesterol showed an increase (*p *> 0.05 for all parameters).

Consistent improvement in all serum lipid parameters were observed in the GA-treated rats relative to the control (*p *> 0.05) (Figure 4). Mean serum TAG showed a 14.73% reduction (control, 1.29 ± 0.31 mmol/L; treated, 1.10 ± 0.27 mmol/L) while that of total cholesterol charted a reduction of 12.99% (control, 3.31 ± 0.60 mmol/L; treated, 2.88 ± 0.43 mmol/L) and that of LDL-cholesterol a 36.96% reduction (control, 1.38 ± 0.34 mmol/L; treated, 0.87 ± 0.27 mmol/L). HDL-cholesterol on the other hand was elevated by 11.85% (control, 1.35 ± 0.19 mmol/L; treated, 1.51 ± 0.47 mmol/L). Serum FFA also exhibited a similar trend of improvement with a reduction of 8.51% in the treated group (control, 0.47 ± 0.07 mmol/L; treated, 0.43 ± 0.07 mmol/L).

### GA treatment reduced tissue lipid deposition

**Figure 5:**
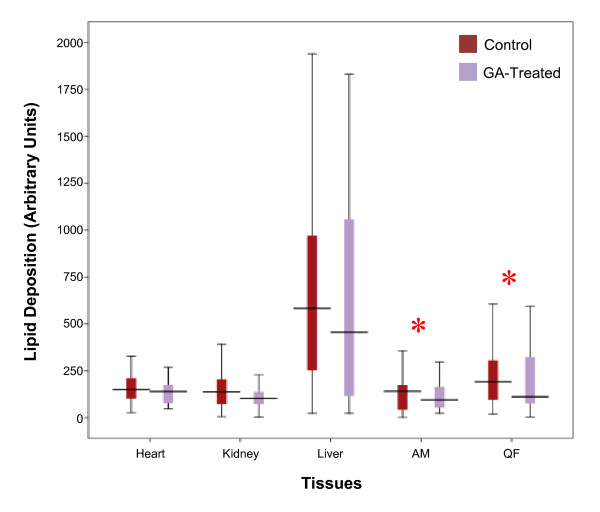
**Levels of lipid deposition in non-adipose tissues**. Sections of ORO-stained tissues were converted to a grayscale each time for lipid staining quantification. * denotes *p *< 0.05.

**Figure 6:**
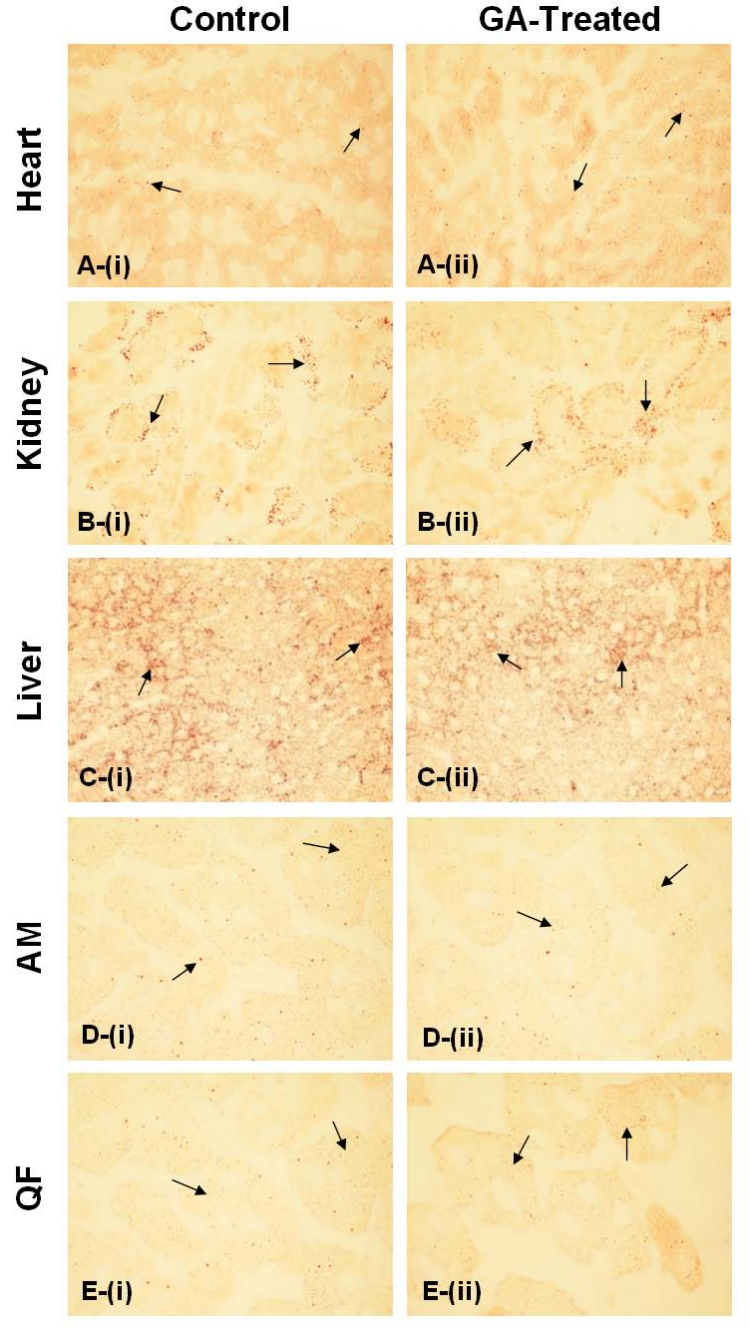
**ORO-stained tissues**. Representative sections of ORO-stained (A) heart, (B) kidney, (C) liver, (D) AM and (E) QF in (i) control and (ii) treated rats at 400× magnification. Distinct spots of ORO-stained lipid were observed across all tissue sections with considerable heterogeneity in lipid content between tissues. Arrows indicate examples of lipid droplets.

Lipid deposition demonstrated a decrease across all studied tissues in the GA-treated group (Figure 5). Levels of lipid deposition was highest in the liver and recorded a 21.86% decrease (control, 582.44 (23.50–1939.66) AU; treated, 55.14 (23.13–1830.91) AU) following GA treatment. The kidney demonstrated a 25.11% decrease (control, 137.54 (11.55–392.10) AU; treated, 103.00 (13.10–228.00) AU). No significant difference between the control and treated groups were observed in both tissues (*p *> 0.05). Among the muscles, the QF and the AM showed significantly reduced lipid deposition in the GA-treated group relative to the control (*p *< 0.05), with a decrease of 42.21% and 33.96% in each tissue respectively (QF: control, 191.28 (28.85–606.17) AU; treated, 110.54 (12.21–594.28); AM: control, 141.29 (11.77–356.51) AU; treated, 93.31 (22.69–297.13) AU). Lastly, lipid deposition in the heart showed a non-significant 6.74% decrease (control, 149.56 (26.58–327.91) AU; treated, 139.48 (47.74–268.54) AU). Sections of these tissues are depicted in Figure 6.

### GA treatment did not induce an increase in systolic blood pressure

**Figure 7:**
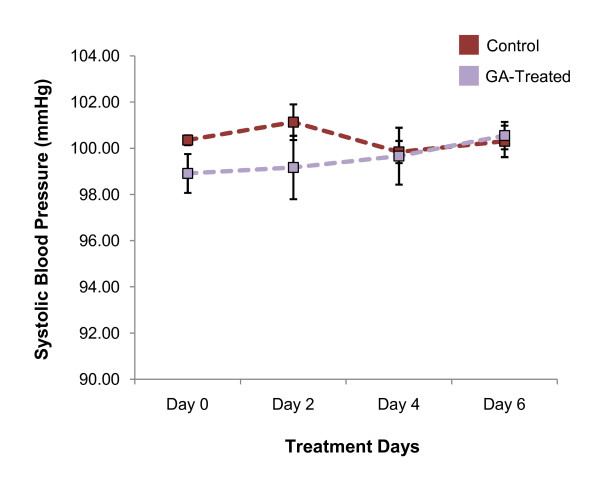
**Evaluation of systolic blood pressure**. Day-to-day mean systolic blood pressure (mmHg) of control and treated rats over the duration of treatment. No significant difference was observed in each of the days between both groups and within each group (*p *> 0.05).

Systolic blood pressure of control and GA-treated rats fluctuated within a narrow range throughout the duration of treatment (Figure 7). Systolic blood pressure of the control and GA-treated rats were compared on Days 0, 2, 4 and 6 of the treatment duration. No significant difference (*p *> 0.05) in mean systolic blood pressure was observed between the control and treated groups and within each group on each of these days.

## Discussion

IR has been recognized as the central component of the MetS which is associated with hyperinsulinaemia, glucose intolerance, dyslipidaemia and visceral obesity [4]. With the onset of IR, the activity of LPL, a key regulator of lipoprotein metabolism that is subject to insulin regulation, has been reported to be reduced both in the adipose tissues and muscles [18,19]. Insulin has been implicated in the biosynthesis of LPL [10] where the insulin-signaling pathway activates the class of nuclear receptors known as the peroxisome proliferator-activator receptor (PPAR). The isoforms of these, PPARα and PPARγ, then bind to the peroxisome proliferator respondse element (PPRE) at the LPL gene promoter to up-regulate LPL expression [20].

In this study, inhibition of 11β-HSD1 by GA could not account adequately for the observed increase in tissue LPL expression. Despite the inhibitory effects of glucocorticoids on LPL protein synthesis and mRNA levels, such observations were only observed in the adipose tissues [21]. Therefore, the induction of LPL expression in this study points to a separate mode of action of GA where GA is postulated to activate the PPAR class of nuclear receptors. This is based on the consistency of several findings, where (i) triterpenoids have been reported to lead to the transactivation of PPAR-γ [22,23] and more importantly (ii) PPAR-α and -γ agonists have been shown to reduce the expression and activity of 11β-HSD1 [24]. Thus, GA, both a triterpenoid and an 11β-HSD1 inhibitor may act as a ligand to the PPAR. Interestingly, 11β-HSD1 knock-out mice also show an elevation of PPAR-α mRNA. PPAR-α is physiologically induced by glucocorticoids and its elevation following 11β-HSD1 inhibition may have arisen from increased circulating plasma corticosterone due to impaired 11β-HSD1-mediated negative feedback upon the hypothalamic-pituitary-adrenal axis [14]. This increased PPARα may then act in return to up-regulate LPL. Thus, GA-mediated activation of the PPAR class of nuclear receptors may be direct or indirect.

PPAR-α plays a key role in regulating pathways of β-oxidation and is expressed abundantly in tissues metabolizing high amounts of FFA, such as the liver, kidney, heart and muscles while PPAR-γ is expressed primarily in the adipose tissues where it triggers adipocyte differentiation and lipogenesis [25,26]. With reference to Figure 1, increased LPL expression was consistently higher in tissues characterized by high PPAR-α expression (QF, AM, kidney and liver) as compared to tissues in which PPAR-γ predominates (VAT and SAT). One exception however was seen in the heart that has a relative LPL expression comparable to that of the adipose tissues. Such discrepancy may be due to the lower distribution of GA into the heart as compared to all other tissues examined in this study [27]. The selective pattern of tissue LPL induction suggested that GA exhibits greater potency in activating PPAR-α than PPAR-γ. Such pattern of tissue LPL induction have been advocated for the correction of visceral obesity as it could lead to the competitive delivery of FFA away from the more pathogenic visceral fat depot to other less pathogenic depots [28].

This postulation was then confirmed in the study through the measurement of adipocyte size in both control and GA-treated rats. With reference to Figure 2, size of adipocytes exhibited a decrease in both the VAT and SAT following GA treatment. The overall results have therefore shown that by reducing visceral fat accumulation, GA has the potential to counteract the very fundamental abnormality that contributes to the development of the MetS, i.e. visceral obesity [8].

Accompanying the increase in tissue LPL expression and decrease in adipocyte size was the consistent improvement in serum lipid parameters of the GA-treated rats relative to the control; with a reduction in serum FFA, TAG, total cholesterol and LDL-cholesterol and elevation of HDL-cholesterol. The GA-induced decrease in serum FFA appears to be of critical importance due to the role of FFA in initiating the development of IR, β-cell dysfunction and dyslipidaemia [5,7]. The observed decrease in serum FFA may be attributed to increased tissue uptake. Berthiaume *et al. *[29] has demonstrated that inhibition of 11β-HSD1 is associated with a concomitant increase in protein content of plasma membrane fatty acid-binding protein (FABP_pm_) that facilitates the entry of FA into cells. In addition, PPAR-α agonists have also been reported to induce the activities of fatty acid transporter protein (FATP) and acyl-CoA synthetase [30], where the former mediates FFA uptake and the latter is involved in the activation of FFA that then facilitates its β-oxidation [31,32]. Activation of acyl-CoA synthetase therefore promotes the oxidation of FFA to prevent the saturation of cellular FA binding and transport [32]. This supports further the decrease in lipid deposition in the studied tissues despite increased FFA uptake.

The decrease in serum TAG did not appear secondary to the reduction in serum FFA and may be mostly attributed to the action of GA. Inhibition of 11β-HSD1 has been shown to reduce hepatic VLDL secretion [29] which may have been driven by increased hepatic FFA oxidation due to the induced expression of fat-catabolizing enzymes [14]. Hepatic VLDL secretion is regulated by the amount of lipids available for the assembly of VLDL [29]. Extracellular FFA entering the liver is either oxidized or esterified to form a cytosolic pool of TAG; the TAG required for VLDL assembly is recruited from this pool. Physiologically, extracellular FFA acts to boost VLDL secretion by expanding the size of this intrahepatic TAG pool [33]. With increased FA oxidation however, the drive for the VLDL assembly pathway is subsequently attenuated.

The increase in HDL-cholesterol following GA administration may be due to the increased production of apo A-I, the major protein of HDL that is subjected to accelerated catabolism in the MetS [7]. Apo A-I mRNA has been shown to be significantly elevated in 11β-HSD1 knock-out mice [14] and following PPAR-α activation [30]. Since the rate of HDL synthesis is dependent on the production of apo A-I [34], this has been speculated as the HDL-increasing mechanism of GA.

Despite lacking benefits of increased LPL expression in this study, such LPL induction by GA may be pivotal in the amelioration of lipid parameters in dyslipidaemic subjects. In the lean rats employed in this study, the serum TAG measured after a 12-h fast reflects only the VLDL fraction. Serum chylomicrons have a half life of 13–14 minutes and would be cleared from circulation within this fasting period [35]. In dyslipidaemic subjects however, hypertriglyceridaemia is attributed to the prolonged retention of both chylomicrons and VLDL due to inhibited lipolysis of both particles following decreased LPL levels [8]. Therefore, in the dyslipidaemic state, induced LPL may contribute to the increased clearance of such lipoproteins to reduce serum TAG. Furthermore, the development of small, dense LDL and the reduction in HDL seen in the dyslipidaemic state are attributed to CETP-mediated lipid exchange between both lipoprotein particles and TAG-enriched VLDL particles. Such exchange is substrate-rather than enzyme-driven [36]. The increased catabolism of TAG-enriched VLDL by LPL may thus serve to positively re-modulate HDL and LDL profile in dyslipidaemia.

The observed decrease in lipid deposition across all studied tissues may be consequential of increased lipid oxidation in these tissues following GA administration. Previous reports have suggested that increased tissue lipid content in the obese state is related to decreased activity of oxidative enzymes [12]. In this study, increase in enzymes of β-oxidation such as acyl-CoA synthetase, mitochondrial carnitine palmitoyltransferase-I and acyl-CoA oxidase were postulated to be induced through (i) direct activation of PPAR-α by GA, (ii) increased expression of PPAR-α following inhibition of 11β-HSD1 [14], or (iii) activation of PPAR-α by LPL-generated FFA that serves as natural PPAR-α ligands [37]. All the enzymes aforementioned carry a PPRE in the promoter region [30,38,39]. The last postulation showed consistency with the results of the study where significant decrease in lipid deposition was observed in the AM and QF, in agreement with their higher increase in LPL expression compared to all other tissues. In addition to the current study, Berthiaume *et al. *[29] has also demonstrated that inhibition of 11β-HSD1 was associated with a reduction in tissue TAG content and increased FFA oxidation.

TAG is present in all cell types and intracellular storage of these neutral lipids occurs within lipid droplets. The adipose tissue and liver are the principal stores of TAG, explaining the high levels of lipid deposition in the liver, while other cell types store small quantities of these. Tissue TAG storage occurs in any quantities, and in the liver for example, TAG storage may range up to 10-fold [33]. This explains the large range of lipid deposition in tissues as observed in the study.

Obesity and T2DM has been associated with tissue lipid accumulation and such ectopic TAG accumulation, also known as tissue steatosis, is implicated in the impairment of insulin signaling [12,13]. These lipotoxic effects are not exerted by TAG itself, but through TAG-derived bioactive lipid metabolites such as long chain fatty acyl-CoA, diacylglyerol and ceramide that activate several serine kinases to block insulin signal transduction [26]. In addition, lipid accumulation in the pancreatic islets would further impair insulin secretion where both ceramide and the nitric oxide generated from surplus unoxidized FFA induces β-cell apoptosis in the pancreatic islet [5]. T2DM has been postulated to only develop in such setting of concurrently occurring IR and β-cell failure [40]. With the demonstrated ability of GA to reduce tissue TAG accumulation therefore, GA exhibits the potential to revert such lipotoxicity exerted by tissue TAG excess and thereby serve to prevent the onset of T2DM.

The analysis of systolic blood pressure was conducted to determine the occurrence of the reported side effects of GA intake. Chronic administration of GA has been associated with the development of pseudoaldosteronism, of which includes symptoms such as electrolyte imbalance and increased blood pressure. This results from the non-selective nature of both GA and GE that inhibits not only 11β-HSD1 but also 11β-HSD2 [17]. In this study, one week administration of GA did not induce an increase in systolic blood pressure. The positive effects arising from the inhibition of 11β-HSD1, such as modulation of serum lipid, that is more readily observable compared to the side effects arising from the inhibition of 11β-HSD2, such as an increase in blood pressure, is possibly due to different potency of GA in inhibiting the two isoforms of the enzyme. Shimoyama *et al. *[41] has reported that GE, the active metabolite of GA, is more effective in inhibiting 11β-HSD1 than 11β-HSD2. The IC_50 _for the two enzymes are 0.09 μM and 0.36 μM respectively. This may signify that the impact of GA on systolic blood pressure may be observed if treatment duration was prolonged, or by increasing the treatment dosage within the same duration. Nevertheless, Quaschning *et al. *[42] has demonstrated that the use of aldosterone and endothelin receptor antagonists could normalize GA-induced blood pressure. The combinatorial use of GA and such antagonists may therefore represent a new therapeutic approach for patients with MetS; allowing patients to harbour the benefits from GA itself and simultaneously eliminating the possible side effects.

## Conclusion

Daily oral administration of 50 mg/kg of GA for a week led to increased LPL expression predominantly in the non-adipose tissues, with significant increase in the QF. Together with the reduction in size of adipocytes in both the VAT and SAT, this may suggest that GA could divert FFA away from the pathogenic visceral depot to the oxidative tissues, thus curbing visceral obesity. GA also modulated serum lipid and the consistent pattern of improvement of each lipid parameter; namely, serum FFA, TAG, total cholesterol, HDL-cholesterol and LDL-cholesterol, points to the ability of GA to cause a beneficial shift to a less atherogenic lipid profile. The decrease in tissue lipid deposition across all the non-adipose tissues studied indicated that lipid did not accumulate in these despite increased LPL expression, possibly due to an accompanying increase in β-oxidation. GA may therefore retard the development of IR associated with tissue steatosis.

## Methods

### Animals and treatment

The use and handling procedure of animals in this research project had been approved by the Monash University Animal Ethics Committee (AEC Approval Number: SOBSB/MY/2007/22). 16 male *Rattus norvegicus *Sprague-Dawley rats weighing between 160–200 g were supplied by Universiti Malaya Animal House (Malaysia) and were housed individually in polypropylene cages in a room kept at 23°C on a 12-h light: 12-h dark cycle (lights on at 0800 hours). The rats were randomly segregated into two groups of eight; representing the control and GA-treated groups. The GA-treated group was given 50 mg/kg of GA daily per oral (p.o.) while the control group given tap water without GA. All animals were fed *ad libitum *with free access to standard rat chow (Glenn Forrest Stockfeeder, Australia) and drinking water for the one week duration of treatment.

### Systolic blood pressure measurement

Systolic blood pressure was measured by tail cuff plethysmography using the NIBP controller (ADInstruments, Australia). Conscious rats were placed into a plastic restrainer and a tail-cuff with a pulse transducer was applied onto the tail. The tail was heated using a table lamp. Rats were allowed to habituate to the procedure for 7 days prior to start of the experiment. The recording and determination of blood pressure were performed using the Chart recording software and a final reading was averaged out from at least 10 consecutive readings. This procedure was performed every alternate day.

### Blood and tissue sampling

At the end of the treatment period, all rats were humanely sacrificed between 0800 to 1000 hours on the 8^th ^day of treatment after a 12-h fast. All rats were anaesthetized via intraperitoneal injection of 150 mg/kg of sodium pentobarbital (Nembutal) prior to exsanguination. Blood was drawn from the cardiac ventricle via the apex and was centrifuged at 12,000 × g for 10 minutes. The resulting serum supernatant was then rapidly aliquoted into microtubes and kept frozen at -80°C until required for analysis. The seven tissues of interest; heart, liver, kidney, AM, QF and VAT and SAT were promptly harvested, all of which were placed into individual cryovials (Nalgene, USA) and immediately snap-frozen in liquid nitrogen. These were then stored at -80°C until required for analysis. In addition, a fraction of VAT and SAT were immersed in 10% neutral-buffered formalin in individual universal bottles for histological analysis.

### Plasma lipid parameters

Total cholesterol, TAG and FFA were measured with Randox CH200 Cholesterol kit (Randox, UK), Wako Triglyceride E kit (Wako, Japan) and Randox FA115 Non-Esterified Fatty Acids kit (Randox, UK) respectively. To determine the level of HDL-cholesterol, HDL-cholesterol was first separated from the LDL and VLDL fraction by precipitation of the latter two using the Randox CH203 HDL Precipitant (Randox, UK), followed by a cholesterol assay using the Randox CH200 Cholesterol kit (Randox, UK). LDL-cholesterol was calculated using the Friedewald formula, using the levels of total cholesterol, TAG and HDL cholesterol obtained [43].

### RNA extraction and cDNA synthesis

Total RNA extraction of the heart, liver, kidney, AM and QF was performed using the Qiagen RNeasy Mini Kit (Qiagen, USA) while that of the VAT and SAT with the Qiagen RNeasy Lipid Tissue Mini Kit (Qiagen, USA). RNA purity was performed by measuring the absorbance of the diluted RNA at 260 and 280 nm. RNase-free DNase treatment was performed using Promega RQ1 RNase-free DNase (Promega, USA) and cDNA synthesis was performed using the Qiagen Omniscript Reverse Transcriptase kit (Qiagen, USA).

### Real time reverse transcription polymerase chain reaction (qRT-PCR)

The expression of LPL was determined by qRT-PCR using the LPL forward and reverse primers 5'-CAGCAAGGCATACAGGTG-3' and 5'-CGAGTCTTCAGGTACATCTTAC-3' and the probe 5'-(6-FAM) TTCTCTTGGCTCTGACC (BHQ1)-3' that are specific for *Rattus norvegicus *LPL mRNA [GenBank: BC081836] and normalized to the β-actin (BAC) gene with the forward and reverse primers 5'-GTATGGGTCAGAAGGACTCC-3' and 5'-GTTCAATGGGGTACTTCAGG-3' and the probe 5'-(TET) CCTCTCTTGCTCTGGGC (BHQ1)-3' specific for *Rattus norvegicus *BAC mRNA [GenBank: BC063166]. The comparison of LPL expression between control and GA-treated rats were performed using the Comparative Ct (ΔΔCt) Method, with BAC as reference, GA-treated group as target and control group as calibrator. Agarose gel electrophoresis was carried out on amplicons generated from qRT-PCR reaction to ensure primer specificity.

### Tissue lipid staining

Frozen tissues were cut into small cubes of approximately 5 × 5 × 5 mm on and embedded using the Optimal Cutting Temperature (OCT) Compound (Leica, Germany). Cryosectioning was performed at a temperature of -25°C where the embedded tissues were sectioned into 5 μm slices and adhered onto glass slides. Staining with Oil Red O (ORO) was performed in accordance to Koopman *et al. *[44] and captured at 400× magnification. Lipid deposition was quantified as specified by Goodpaster *et al. *[12]. Images were transferred to Image J software and converted to grayscale. Threshold for the intensity of staining was adjusted in order to pick up only the droplets of lipid; the full-range being from 0 to 255 arbitrary units (AU), where 0 represents complete staining and 255 represents no staining. For lipid quantification, pixels with intensities of ≤ 150 ± 30 AU were quantified. Lipid deposition were expressed in AU and calculated as:



Eight contiguous views per tissue section were captured and analyzed for the lipid content. The level of lipid deposition of each tissue section was calculated as the average of these eight values.

### Morphometric analysis of adipocytes

Adipose tissues that were fixed in 10% neutral-buffered formalin as aforementioned were processed by a Leica TP 1020 Automatic Tissue Processor and embedded in paraffin. 5 μm thick tissue sections were then stained with haematoxylin and eosin (H&E) followed by the measurement of the size of 100 adipocytes (μm^2^) per field view per tissue section at 100× magnification.

### Statistical analysis

Statistical analysis of LPL expression was performed using the Relative Expression Software Tool (REST^©^) MCS Beta 2006 while that of all other parameters was performed using the Statistical Package for the Social Sciences (SPSS) Version 16.0. Data distribution was analyzed using the Kolmogorov-Smirnov test. Parametric data were then analyzed with independent t-test and are presented as mean ± standard error while non-parametric with Mann-Whitney U-test and are reported as median (minimum – maximum). In all analyses, a *p*-value ≤ 0.05 was considered significant.

## Abbreviations

11β-HSD: 11β-hydroxysteroid dehydrogenase; AM: abdominal muscle; CETP: cholesteryl ester transfer protein; FFA: free fatty acids; GA: glycyrrhizic acid; GE: glycyrrhetic acid; H&E: haematoxylin and eosin; HL: hepatic lipase; HDL: high-density lipoprotein; IR: insulin resistance; LDL: low-density lipoprotein; LPL: lipoprotein lipase; MetS: metabolic syndrome; ORO: Oil Red O; PPAR: peroxisome proliferator-activator receptor; PPRE: peroxisome proliferator response element; QF: quadriceps femoris; SAT: subcutaneous adipose tissue; T2DM: type 2 diabetes mellitus; TAG: triacylglycerol; VAT: visceral adipose tissue; VLDL: very-low-density lipoprotein.

## Competing interests

The authors declare that they have no competing interests.

## Authors' contributions

WYAL was involved in all bench work, data acquisition, analysis and interpretation and manuscript preparation. YYC had part in the optimization of the qRT-PCR conditions and SYL had part in histological work. SHT, KAK and SNASH participated in the coordination of the study and helped in drafting the manuscript. All authors read and approved the final manuscript.
